# Physiotherapy in Bell’s Palsy Secondary to Acute Otitis Media: A Case Report

**DOI:** 10.7759/cureus.60795

**Published:** 2024-05-21

**Authors:** Shrushti Jachak, Raghumahanti Raghuveer

**Affiliations:** 1 Department of Neurophysiotherapy, Ravi Nair Physiotherapy College, Datta Meghe Institute of Higher Education and Research, Wardha, IND

**Keywords:** acute otitis media, physiotherapy, case report, facial nerve palsy, bell’s palsy

## Abstract

Acute suppurative otitis media can occasionally result in facial paralysis, which calls for prompt diagnosis and treatment. Facial paralysis, a synonym for Bell's palsy, is a condition that causes rapid weakening of one side of the face muscles, leading to drooping of the face on that side. A major factor in determining the course of the condition is rehabilitation through physiotherapy. Here, we present a case of a 26-year-old female who felt discomfort in her left ear on February 21, 2024, but chose to ignore it then. She observed an abrupt deviation in her mouth and visited a rural hospital, where she was admitted. Facial asymmetry was observed during the examination, and she was found to have a grade V on the House-Brackmann scale. A near-normal muscle action was initiated by mime therapy, and proprioceptive stimulation was given by facial proprioceptive neuromuscular facilitation along with electrical stimulation. All these approaches benefited the patient in a significant manner.

## Introduction

The facial nerve, the seventh nerve, is one of the most critical nerves in the human body, which regulates facial expression and movement. Bell's palsy is thought to be triggered by an immune system attack on the nerve that controls facial movement, and diabetes, hypertension, injuries, toxins, multiple sclerosis, Lyme disease, sarcoidosis, Guillain-Barré syndrome, myasthenia gravis, Ramsay Hunt syndrome, and ear infections subsequently are linked to it. Bell’s palsy has been determined to be most common in adults aged 15 to 45. As a result, individuals may suffer a considerable psychological toll [1,2]. The yearly incidence is around 23 per 100,000 individuals or roughly one in 60 to 70 people throughout their lives. Facial nerve palsy is a prevalent clinical mononeuritis, with incidence rates ranging from 11.5 to 53.3 per 100,000 person-years, depending on the country [3]. In recent times, facial nerve palsy has become an infrequent consequence of acute otitis media, with an approximate incidence of 0.005%. Prior to the development of antibiotics, this consequence was extremely prevalent, with an estimated prevalence of 0.5-0.7% [4]. Among the most pervasive symptoms of Bell's palsy are abnormal movements of the muscles that control facial expressions, such as blinking, smiling, or shutting the eyelid. Numbness on one side of the face, drooling of saliva, excessive lacrimation, and headache are also seen. In the anterior two-thirds of the tongue, the taste can also deteriorate. Facial asymmetry, hemifacial spasm, crocodile tears, corneal ulceration, and irreversible vision disability can also be seen. Most Bell's palsy patients recover partially without any intervention in less than two to three weeks of the beginning of symptoms and entirely within three to four months of the commencement of symptoms [5]. Although paralysis usually resolves on its own, it might have long-term consequences that need to be resolved. Otitis media, an ear infection, can also cause facial palsy. Changes in the middle ear microenvironments, such as high pressure, osteitis, or acute inflammation, are likely to be the cause of facial nerve paralysis in acute suppurative otitis media. These changes may have a direct impact on the physiology of the facial nerve [4]. Prednisone and other corticosteroids are anti-inflammatory drugs with a lot of potency. These medicines are hazardous for pre-existing diseases. Consequently, successful supplementary therapy would aid in the treatment's success [6]. Physical therapy also fastens the recovery. Mime therapy, which uses non-verbal expressions to convey a story, can improve the patient’s condition by promoting symmetry of the face at rest and while moving. The use of the face proprioceptive neuromuscular facilitation (PNF) approach reduces facial impairment by altering neuromuscular education via facilitation. Electrical muscle stimulation also helps in the stimulation of muscles and its normal functioning. General measures such as wearing goggles, performing repeated muscle activity, and maintaining proper hygiene also aid in recovery. In our case, we used electrical stimulation in conjunction with facial PNF and mime therapy, which is an interesting treatment approach and enhances the patient's adherence to the treatment.

## Case presentation

Patient information

A 26-year-old female suddenly experienced left ear pain on February 21, 2024, which she ignored. On February 22, when she woke up, she could not brush her teeth properly and experienced weakness on the left half of her face; she also could not hold the water in the mouth while brushing her teeth. The patient noticed facial asymmetry. On February 22, she visited the local physician, where the physician referred the patient to a rural hospital, where the patient was assessed and the diagnosis was made. Following admission, the medical management was initiated. The patient was admitted for 10 days to the ear-nose-throat (ENT) ward.

Clinical findings

**Figure 1:**
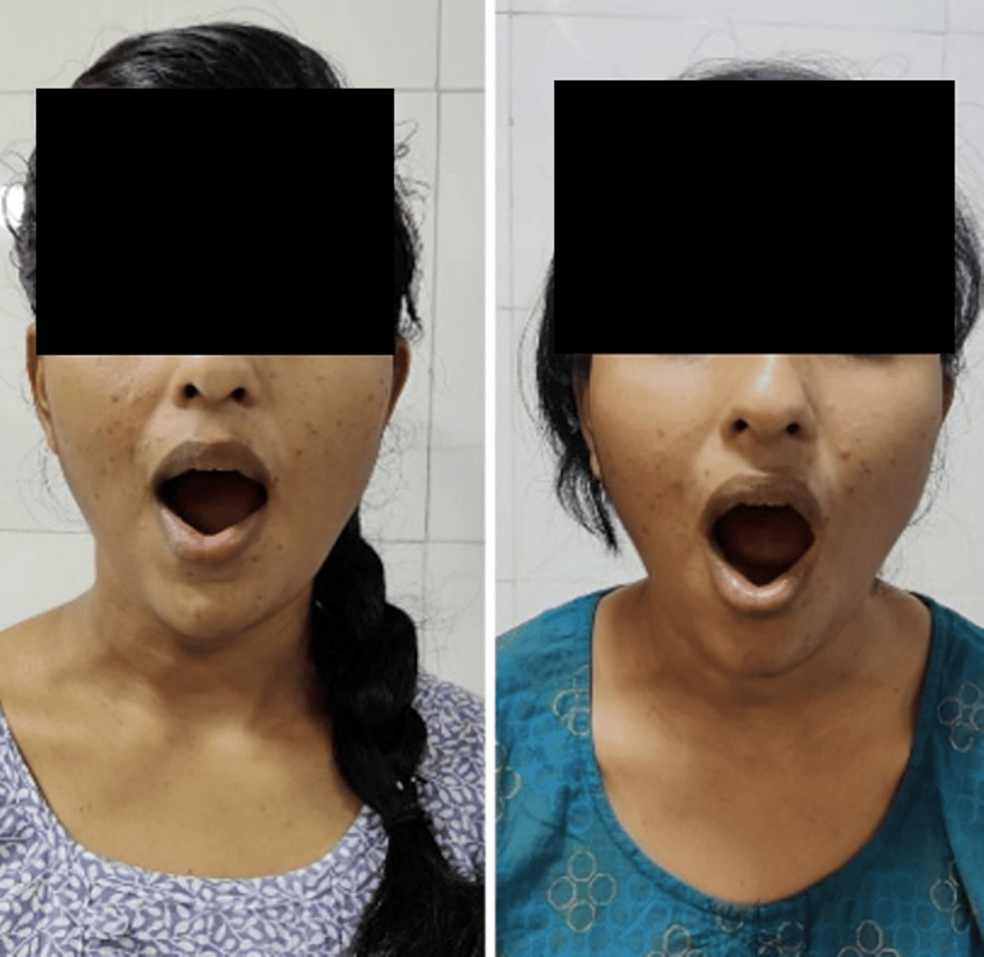
Orbicularis oris pre and post rehabilitation respectively Picture denoting Orbicularis oris muscle action pre and post-rehabilitation respectively

**Figure 2:**
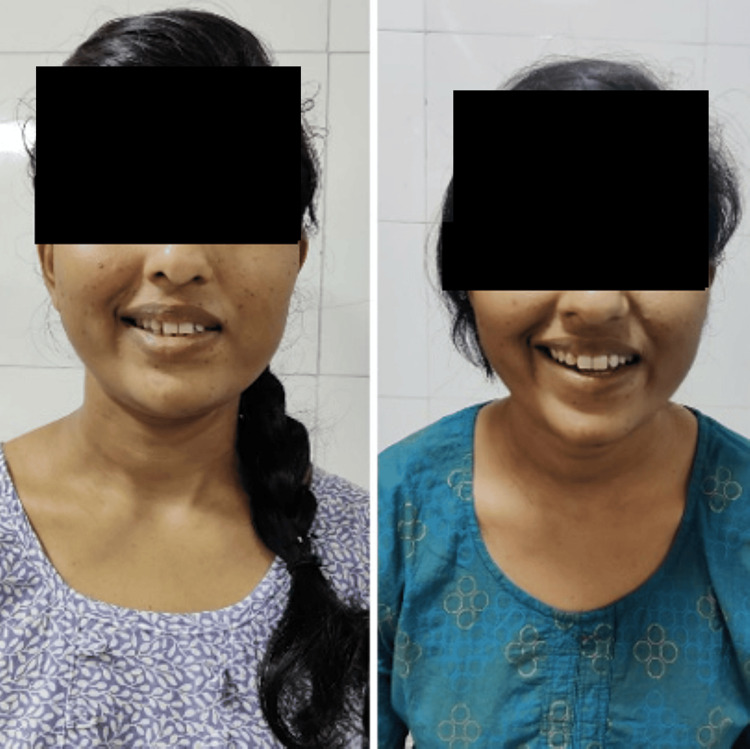
Masseter muscle action pre- and post-rehabilitation

**Figure 3:**
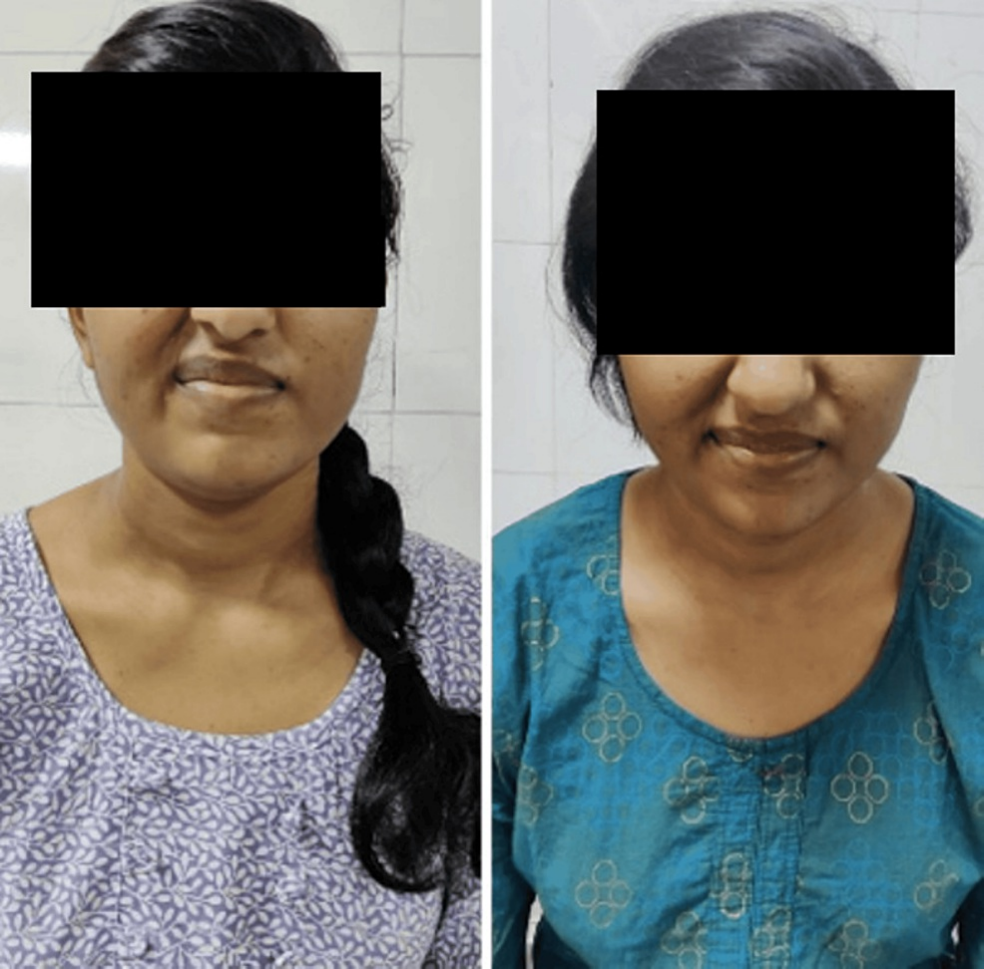
Nasalis muscle action pre- and post-rehabilitation

**Figure 4:**
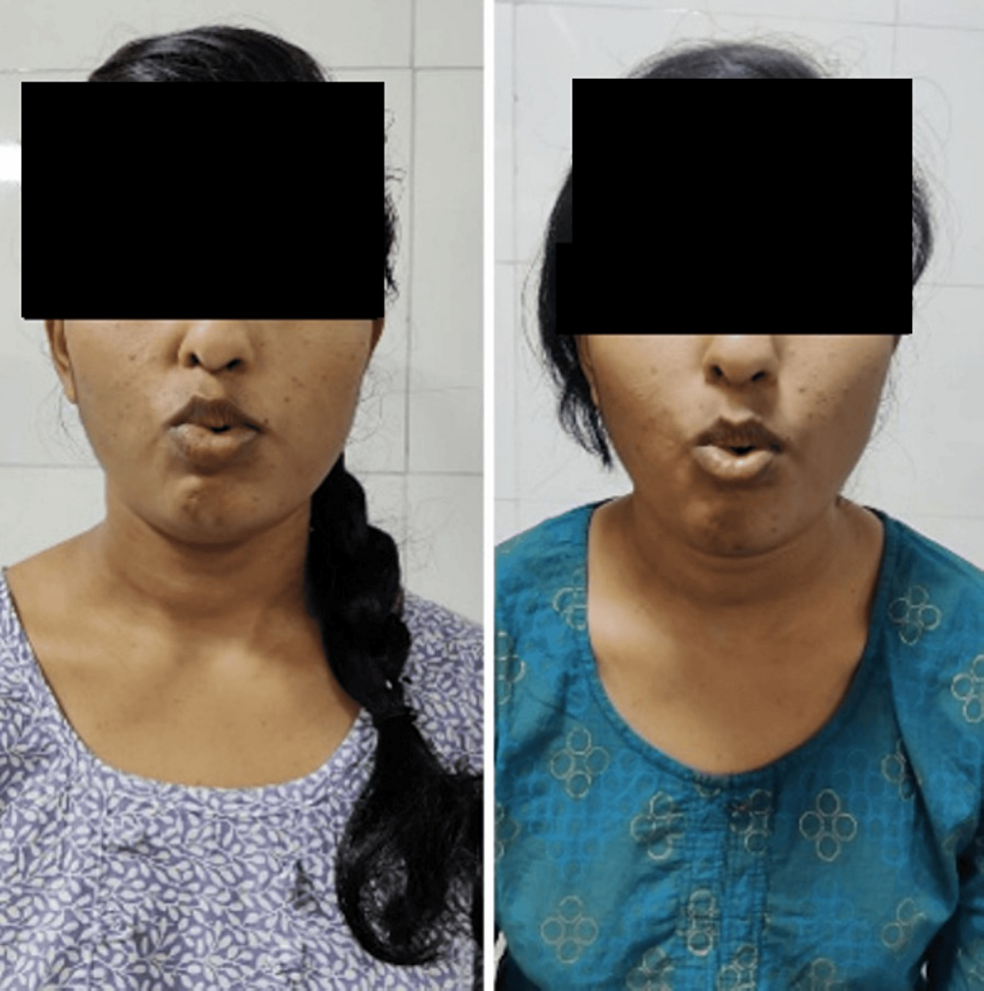
Buccinator muscle action pre- and post-rehabilitation

**Figure 5:**
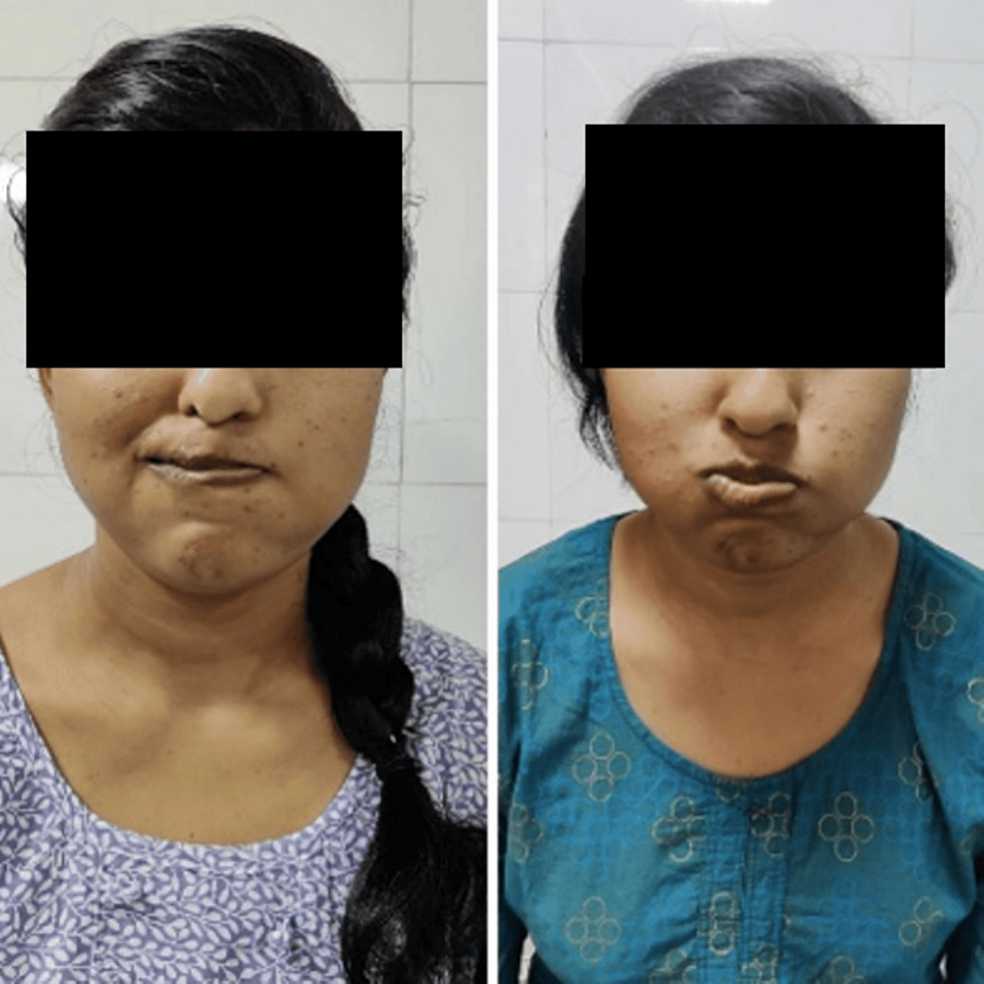
Buccinator muscle action pre- and post-rehabilitation

**Figure 6:**
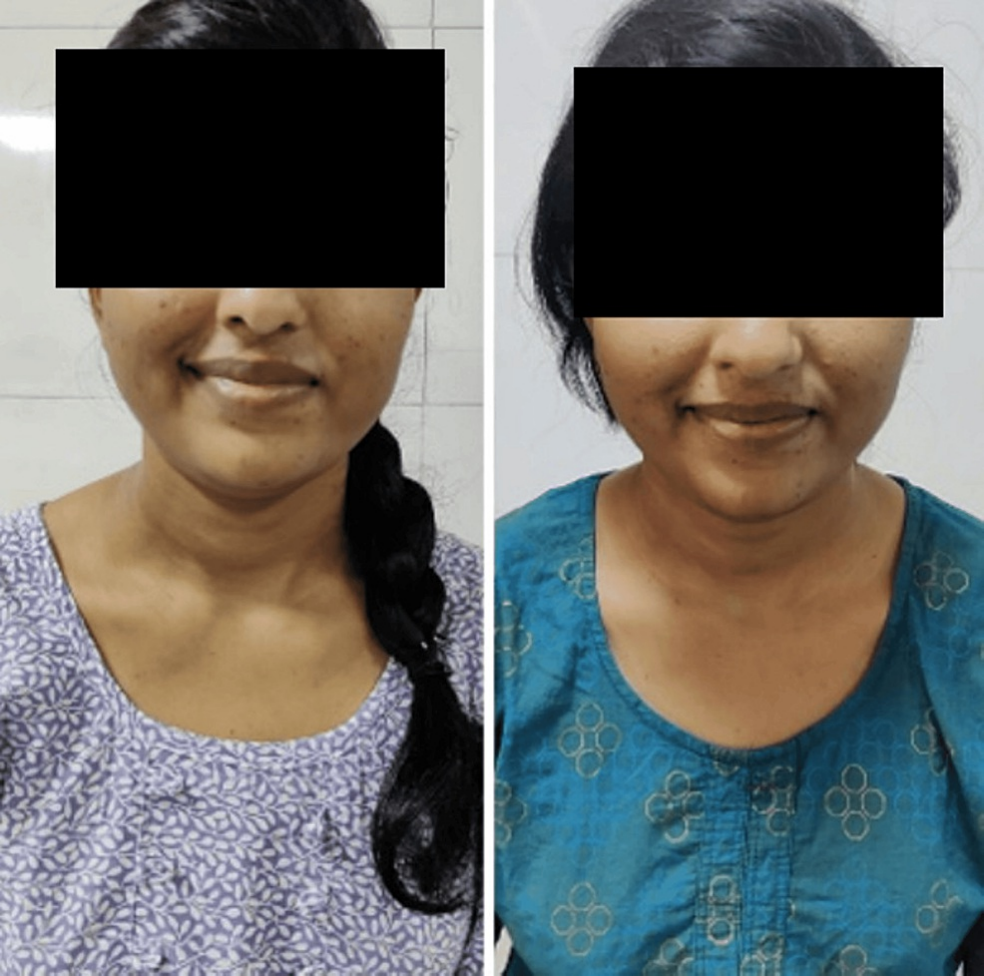
Zygomaticus major muscle action pre- and post-rehabilitation

**Figure 7:**
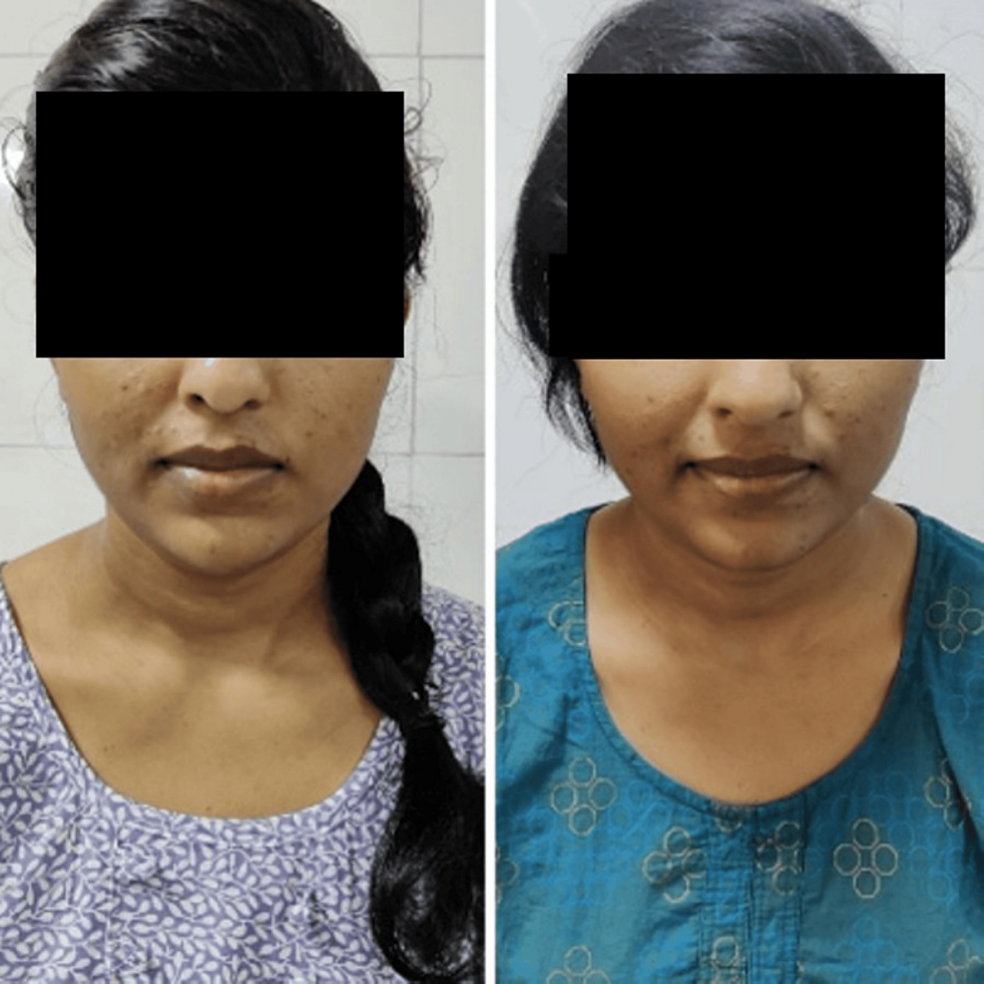
Frontalis muscle action pre- and post-rehabilitation

**Figure 8:**
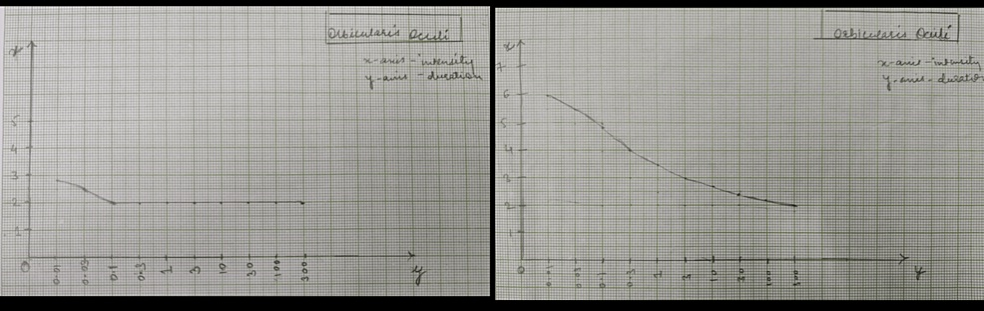
Strength-duration curve of the orbicularis oculi muscle pre- and post-rehabilitation

An informed oral consent was taken from the patient. On inspection, facial asymmetry was seen with left-sided affection; the nasolabial fold was absent, the angle of the mouth was dropped, forehead wrinkle was not noticed, and eye closure was incomplete. The facial excursion was significantly reduced. Bell’s phenomenon was present. On examination, the House-Brackmann score was 5, indicating severe dysfunction. The strength-duration curve was plotted for every facial muscle. At the start of the treatment, a Sunnybrook facial grading system was used, on which the score was 12. A synkinesis assessment questionnaire (SAQ) was also administered to the patient, on which the score was 31. The pictures of the pre-rehabilitation stage and post-rehabilitation are shown in Figures 1-7, and the strength-duration curve pre- and post-rehabilitation is shown in Figure 8.

Timeline

The timeline of the events is listed in Table 1.

**Table 1:** Timeline of the events

Events	Date
Onset of symptoms (ear pain)	February 21, 2024
Onset of facial weakness, incomplete eye closure, dropping of angle of mouth	February 22, 2024
Date of admission	February 22, 2024
Date of discharge	March 2, 2024
Date of physical rehabilitation	March 12, 2024
Date of follow-up	April 20, 2024

Intervention

The patient was diagnosed with left-sided Bell's palsy, for which she was admitted to a rural hospital for 10 days, where she was treated with conservative medical treatment (Tablet amoxicillin, Zerodol, Neurobion Forte, Vertin, Rabemac, Naxdom; ear drop: moxifloxacin; eye drops: Moxi, I-dew; and nose drop: Otrivin). After five days, she was referred to the Neurophysiotherapy OPD, where she was educated about PNF for the face as a home exercise program; electrical stimulation was not started as the nerve was inflamed and was not regenerated, and thus the patient was asked to visit after a few days. On March 12, after 10 days of discharge, she presented to the Neurophysiotherapy OPD again.

A three-week protocol was given to the patient, which included a session of five days per week for 40 minutes every day.

Week 1: The patient was educated about her condition at the start of the physiotherapy session. The importance of the rehabilitation was explained to the patient. Ergonomic advice, such as wearing goggles for eye protection and taping the eye, was given. She was also advised to drink water using a straw. As the patient was educated earlier about the face PNF program, she was started with the same program in OPD, along with mirror biofeedback (10 repetitions x one set) adjunct to the electrical stimulation (interrupted galvanic current, 45 contractions x three sets) [7]. The facial massage was started. Balloon and candle-blowing exercises were also prescribed to her.

Week 2: The face PNF was continued along with the mirror biofeedback (15 repetitions x 1 set) [8], which followed the lymphatic drainage effleurage for the face. Electrical stimulation was continued (interrupted galvanic, 45 contractions x two sets). Oromotor training was started adjunct to the above treatment.

Week 3: PNF was continued, which progressed to the anti-gravity plane with resistance [8], followed by mirror therapy (20 repetitions x one set); oromotor training was also continued. Electrical stimulation was stopped as the improvement was remarkable, which can be seen on strength-duration curve. Mime therapy was started by this time (daily once for one week); it is a very fun therapy, as it involves non-verbal expressions to convey a story.

Follow-up and outcome

The patient was followed up weekly once for one month. There was a significant reduction in facial asymmetry. Post-treatment House-Brackmann grade was II. Post-rehabilitation photos and strength-duration curve are shown in Figures 1-8. Pre- and post-treatment outcome measures are listed in Table 2.

**Table 2:** Outcome measure pre- and post-rehabilitation

Outcome measures	Pre-rehabilitation	Post-rehabilitation
House-Brackmann grade	Grade V	Grade II
Sunnybrook score	12	75
Synkinesis assessment questionnaire score	31	20

## Discussion

Many studies have been conducted that comprised management of physical therapy in addition to conservative medical treatment. Numerous studies used electrical stimulations, PNF techniques, oromotor training, mirror biofeedback, and the latest therapy that was used was mime therapy [9]. Alakram and Puckree conducted a study that aims to explore if electrical stimulation in the early stages of Bell's palsy was safe and effective with 16 individuals who had Bell's palsy for less than 30 days. Adult Bell's palsy patients were divided into control and experimental groups (every second patient). They found that electrical stimulation was shown to be safe but not superior to rapid recovery or multimodal physiotherapy in this trial. It was concluded that the benefit and safety were evident but not superior [10]. We have given the electrical stimulation and found it effective and safe in our patient as well. In conjunction with this study, Tuncay et al. studied how effective electrical stimulation improves clinical and neurophysiologic results in people with Bell’s palsy when used with routine physical therapy. The treatment was given for three weeks. It was seen that patients who soon after the onset of facial weakness (four weeks) underwent daily electrical stimulation for three weeks showed significant results in functional movements of the face and electrophysiologic outcome markers [11]. We also found same results after using the electrical stimulation after 21 days of injury. Mime therapy's effectiveness was investigated in our research. In this study, we also found that mine therapy works well and is very easy to make the patient understand what he has to perform. Beurskens and Heymans conducted a research, which also revealed similar results [10]. Mishra and Sayed used mime therapy adjunct to electrical stimulation, and the results noted were positively convincing. The purpose of this study was to determine how functional abilities and face symmetry were affected in Bell’s palsy patients by mime treatment combined with sensory exercises. Thirty individuals in total were recruited for an interventional study and randomly assigned to three groups (n=10): group A got electrical stimulation in the form of facial exercises, group B got electrical stimulation in the form of mime therapy, and group C got electrical stimulation in addition to sensory exercises and mime therapy. Eighteen sessions, lasting an hour and a half six days a week for three weeks, were given to each group [12]. In a similar case study conducted by Deshpande et al., results showed that face expressiveness was significantly enhanced after a two-week incorporation of the facial muscle PNF, Matrix Rhythm treatment, and traditional facial stimulation. The focus of the study was on the results of combining PNF and Matrix Rhythm Therapy with traditional electrical stimulation. For two weeks, there was also a combination of Matrix Rhythm Therapy with each weekly appointment. Combining traditional facial muscle stimulation, face muscle PNF, and Matrix Rhythm Therapy. Even without adding the Matrix Rhythm Therapy, positive outcomes were reported [13]. This result was in line with our result. This is a distinctive case as a combination of various treatment approaches was used in the management. The results were seen in a short duration of time and were significantly seen through the outcome measures used.

## Conclusions

In our case report, the patient was given a thorough strategy that assisted her in achieving face symmetry and muscular strength. All of the facial excursions seen in the patient were accomplished by the patient. The patient's condition improved significantly, and the patient had no insecurities. Finally, the findings of this case study suggest that a patient with acute otitis media owing to Bell's palsy recovered entirely with conservative therapy and may be clinically implicated in cases of facial nerve palsy, hence benefiting those in need in a community. We used Mime therapy as one of the major interventions. Mime therapy uses facial expressions to tell a story; if a patient is indeed not expressive we, are not able to quantify and identify the recovery of the patient, which is one of the major limitations of this case report. This case report can aid in treating a patient with Bell's palsy secondary to otitis media. This protocol is suggested to treat a patient with a similar condition.
